# Rapid Genetic Algorithm Optimization of a Mouse Computational Model: Benefits for Anthropomorphization of Neonatal Mouse Cardiomyocytes

**DOI:** 10.3389/fphys.2012.00421

**Published:** 2012-11-05

**Authors:** Corina T. Bot, Armen R. Kherlopian, Francis A. Ortega, David J. Christini, Trine Krogh-Madsen

**Affiliations:** ^1^Greenberg Division of Cardiology, Weill Cornell Medical CollegeNew York, NY, USA; ^2^Department of Physiology, Biophysics and Systems Biology, Weill Cornell Medical CollegeNew York, NY, USA; ^3^Institute for Computational Biomedicine, Weill Cornell Medical CollegeNew York, NY, USA

**Keywords:** dynamic clamp, genetic algorithm, cell-specific model, neonatal mouse cardiomyocyte

## Abstract

While the mouse presents an invaluable experimental model organism in biology, its usefulness in cardiac arrhythmia research is limited in some aspects due to major electrophysiological differences between murine and human action potentials (APs). As previously described, these species-specific traits can be partly overcome by application of a cell-type transforming clamp (CTC) to anthropomorphize the murine cardiac AP. CTC is a hybrid experimental-computational dynamic clamp technique, in which a computationally calculated time-dependent current is inserted into a cell in real-time, to compensate for the differences between sarcolemmal currents of that cell (e.g., murine) and the desired species (e.g., human). For effective CTC performance, mismatch between the measured cell and a mathematical model used to mimic the measured AP must be minimal. We have developed a genetic algorithm (GA) approach that rapidly tunes a mathematical model to reproduce the AP of the murine cardiac myocyte under study. Compared to a prior implementation that used a template-based model selection approach, we show that GA optimization to a cell-specific model results in a much better recapitulation of the desired AP morphology with CTC. This improvement was more pronounced when anthropomorphizing neonatal mouse cardiomyocytes to human-like APs than to guinea pig APs. CTC may be useful for a wide range of applications, from screening effects of pharmaceutical compounds on ion channel activity, to exploring variations in the mouse or human genome. Rapid GA optimization of a cell-specific mathematical model improves CTC performance and may therefore expand the applicability and usage of the CTC technique.

## Introduction

Dynamic clamp is a closed-loop hybrid experimental-computational technique that permits probing a living cell with current-clamp perturbations that are calculated functions of instantaneous measurements of the behavior of the cell. As such, dynamic clamp is useful for investigating ion channel function and ionic current dynamics (Dorval et al., 2001; Prinz et al., 2004; Raikov et al., 2004; Bettencourt et al., 2008). The method also allows coupling real cells to computational model cells, and permits hybrid networks containing an arbitrary number of simulated and real cells (Berecki et al., 2005, 2006; Wilders, 2006; Kispersky et al., 2011). Some recent implementations of dynamic clamp have become highly complex (Lin et al., 2010; Kispersky et al., 2011), allowing investigations into questions that are not readily amenable by traditional approaches, such as basic current- and voltage-clamp electrophysiological methods (Lin et al., 2010; Idoux and Mertz, 2011; Kispersky et al., 2011; Madhvani et al., 2011; Nguyen et al., 2011).

One particular research topic that may benefit from dynamic clamp tools is the study of interspecies differences in action potentials (AP) of excitable cells, such as cardiac myocytes. Such interspecies differences can limit the extent to which results from animal models can be extrapolated to human physiology. AP differences, which are largely due to differences in the expression levels and subtypes of ion channels and transporters (Nerbonne, 2004; Kaese and Verheule, 2012; O’Hara and Rudy, 2012), are especially pronounced when comparing human and murine ventricular cardiomyocyte dynamics. The murine AP is much shorter in duration and more triangular than that of a human myocyte. Many anti-arrhythmic drugs and genetic mutations affect the plateau of sustained depolarization in humans; testing their influence only in the mouse – with its qualitatively different plateau morphology – can lead to uncertain interpretations. For instance, a drug that looks promising in a mouse model may not work for a human heart. Given the importance of the mouse as a model organism, novel insights into interspecies differences and techniques to overcome them are valuable.

**Figure 1:**
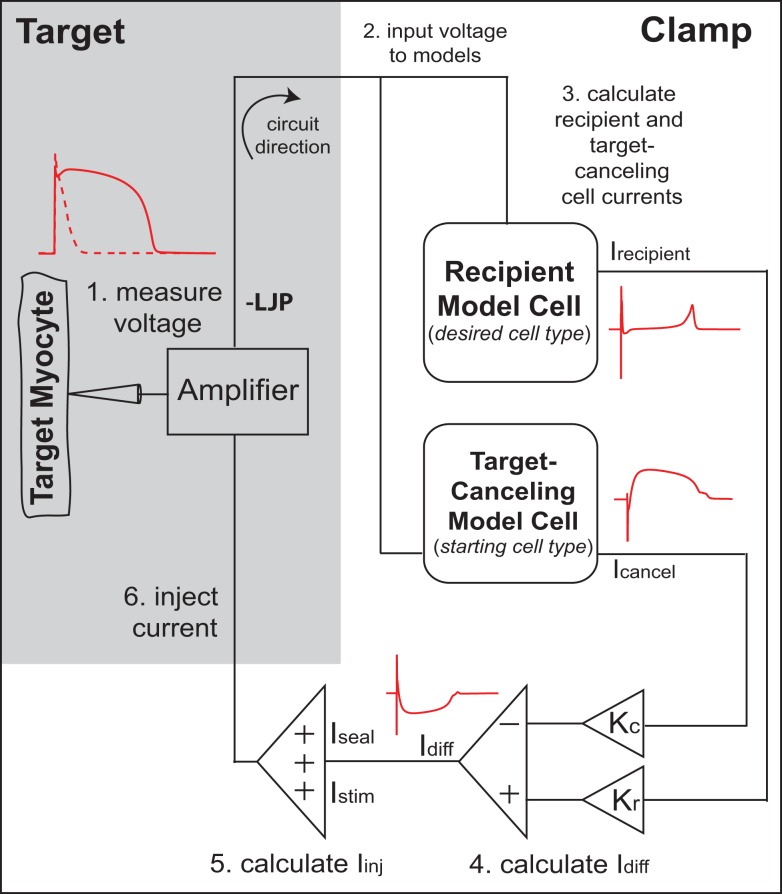
**Cell-type transforming clamp circuit (adapted from Ahrens-Nicklas and Christini, 2009 with permission from Elsevier)**. A real cell (target myocyte) is simultaneously coupled to a target-canceling computational model (a model of the native isolated cell) and a recipient computational model (a model of the desired cell-type, i.e., human or guinea pig), in a closed-loop circuit. A time-dependent current that compensates for the difference between target cell and recipient model cell currents is inserted into the myocyte at each instance of the measured voltage. The myocyte responds to the injected current such that the membrane voltage is transformed to the recipient model. An offset was added to correct the measured voltage for the liquid junction potential (LJP).

It was recently demonstrated that the murine cardiac AP waveform could be anthropomorphized into that of a human-like AP in real-time, through a novel dynamic clamp method known as the cell-type transforming clamp (CTC; Ahrens-Nicklas and Christini, 2009). In the CTC, a computationally calculated current is inserted into the cell in real-time (Figure 1) to compensate for the intrinsic differences between murine and human sarcolemmal currents. In so doing, the CTC anthropomorphizes the membrane potential without action potential clamping it.

Cell-type transforming clamp makes use of a mathematical model to mimic the currents generating the murine AP in order to effectively cancel out the total sarcolemmal current in the measured cell and replace it by the human-like current. CTC therefore assumes a close resemblance between total sarcolemmal current in the mouse myocyte (the target myocyte) and the mathematical mouse model (the target-canceling model cell), and hence a close similarity between their AP waveforms. Because of cell-to-cell variability in AP shape and duration in the real myocyte population (e.g., Babij et al., 1998), such matching constitutes a difficult problem. Indeed, our previously published mismatch-reduction approach of selecting one of nine candidate models based on AP duration (APD; Ahrens-Nicklas and Christini, 2009) can be limited in its ability to reliably match a range of shapes of the murine AP. Hence, a method that allows better fitting of the heterogeneous population of measured murine APs is required. This study uses an optimization technique called a genetic algorithm (GA) to efficiently provide a cell-specific model. GAs represent an optimization technique of simultaneous initial-value parameter modifications, inspired from evolutionary biology principles (Kherlopian et al., 2011). GAs have been used previously in cardiac model studies (Syed et al., 2005; Mathavan, 2009), but we know of no studies that applied a GA during an experiment; such an implementation is complicated by the large parameter space the GA must navigate and the long time scales required to achieve convergence. We have brought to bear a rapid GA optimization by varying only key parameters in a computationally tractable cardiac myocyte model, as well as constraining model run time and GA population size and generation count.

Here we use the CTC to convert neonatal mouse ventricular myocytes, to the morphology of two desired cell models (i.e., recipient models): guinea pig and human. Our findings show that GA optimization of the target-canceling model to match the experimentally measured cell results in more accurate CTC conversion than using candidate model selection relying only on APD. This better performance was particularly striking when using the human recipient cell model. Our study thus presents an improvement to the existing CTC technique, providing the means to automatically tune the target-canceling model to match the target cell, using the GA.

## Methods

### Neonatal mouse cardiomyocyte isolation

All procedures were done in accordance with Weill Cornell Institutional Animal Care and Use Committee regulations. Single ventricular myocytes were isolated from day-2 mouse pup hearts, using a protocol modified from Ahrens-Nicklas and Christini (2009); Brand et al. (2010). Mice were anesthetized via inhalation of isoflurane, their beating hearts surgically removed, and immediately placed into ice-cold 1× ADS buffer, over ice (Brand et al., 2010). Hearts from one litter of pups were pooled, washed once with 1× ADS buffer, and the ventricles isolated and minced. Tissue was transferred into a solution of 1 mg/ml collagenase (Worthington Type II) in 1× ADS buffer with a 10% pancreatin solution (pre-warmed), and incubated with shaking for a 5 min blood wash at 37°C. Supernatant was discarded, 10 ml collagenase solution was placed over the tissue, and incubated with shaking for 20 min. After incubation, tissue was triturated, supernatant was collected, and filtered through a sterile 100 μm cell strainer over 2 ml of horse serum, and centrifuged at 100×*g* for 5 min. Supernatant was discarded and cells were resuspended in 4 ml horse serum, with the falcon tube loosely capped in a 5% CO_2_ incubator at 37°C. This constitutes the first cell collection. Meanwhile, 10 ml collagenase solution was placed over the remaining heart tissue and incubated again, with shaking, for 25 min. The above tissue digestion procedure was repeated for the second cell collection. Both fractions were pooled, and a period of 20–25 min of cell recovery in horse serum, in the CO_2_ incubator was allotted.

Next, myocytes were centrifuged at 100×*g* for 5 min and resuspended in “day of isolation” culture medium. This culture medium consists of a 4:1 mixture of Dulbecco’s Modified Eagle Medium (DMEM) containing 4.5 g/l and medium M199, 5 mM HEPES, 2 mM l-glutamine, and 1× penicillin–streptomycin, pH 7.4 (Brand et al., 2010). Day of isolation culture medium is supplemented with 10% heat-inactivated horse serum and 5% heat-inactivated fetal calf serum.

Cells were pre-plated for 45 min at 37°C to reduce the number of fibroblasts in the final cultures. After pre-plating, viable myocytes were counted using a 0.4% Trypan Blue solution, and cultured into tissue-culture treated dishes at a final concentration of 8 × 10^4^ to 1 × 10^5^ cells/ml to ensure the presence of isolated cells. Cells were kept at 37°C in a 5% CO_2_ incubator, and next day the media was changed to “day 2 of culture” medium. This medium has the same characteristics as “day of isolation” medium described above, except for a lower serum concentration (4% heat-inactivated fetal calf serum). Myocytes were used for electrophysiology studies in the first 24–48 h after culture.

### Electrophysiological recordings

Whole-cell current-clamp recordings were performed at room temperature (22–24°C). Myocytes were superfused with a Tyrode’s solution containing (in mM): NaCl 139, KCl 4, glucose 5.5, MgCl_2_ 1, CaCl_2_ 1, HEPES 10, pH 7.4. Pipette solution contained (in mM): KCl 143, Mg_2_ATP 5, EGTA 0.05, HEPES 10, MgCl_2_ 1, CaCl_2_ 0.025, pH 7.1. Osmolality recorded with a Vapro 5520 vapor pressure osmometer averaged 291 ± 2 mmol/kg for the intracellular solution and 298 ± 1 mmol/kg for the extracellular solution.

Pipettes pulled from 1.5 mm glass capillary tubes (Sutter Instrument, Novato, CA, USA) had a mean resistance of 5.2 ± 0.2 MΩ (mean ± SE) in solution, and offset potentials were measured and corrected. Recordings were performed using the Real-Time eXperiment Interface (RTXI; http://www.rtxi.org), a real-time Linux-based experimental control software system developed in our laboratory (Dorval et al., 2001; Bettencourt et al., 2008; Lin et al., 2010), and an A-M Systems (Sequim, WA, USA) model 2400 patch-clamp amplifier.

Cell capacitance was measured in voltage-clamp mode, by adjusting amplifier whole-cell compensation knobs (which measure membrane capacitance and access resistance) to minimize transients resulting from the application of a 10 mV square wave of 10 ms duration (Ahrens-Nicklas and Christini, 2009). Capacitance was measured for each cell (average value 14.8 ± 0.6 pF) and used in conjunction with model cell capacitance values to scale the CTC currents (Ahrens-Nicklas and Christini, 2009). Bridge balance was used to compensate for the voltage drop across the access resistance, which averaged 3.5 ± 0.8 MΩ. The liquid junction potential (−3 mV, measured by standard procedure; Neher, 1992) was adjusted for as illustrated in Figure 1 above. Patch seal resistance measured for each cell (mean value 5.6 ± 0.6 GΩ) was used to calculate the seal leak current (*I*_seal_) in CTC (Ahrens-Nicklas and Christini, 2009). APs were evoked at a rate of 1 Hz using depolarizing stimuli of 1 ms duration and 0.9 ± 0.04 nA amplitude.

### Data analysis

Resting membrane potential was measured prior to each AP as the membrane potential in the timestep before onset of stimulus current. Mouse myocyte and mouse model APD was measured from the time of onset of the AP upstroke to the time when the potential returned 80% of the way from 0 mV to the resting membrane potential (APD_80_). The duration of CTC converted AP was quantified at 30, 50, and 90% repolarization (APD_30_, APD_50_, and APD_90_) measured from the time of the upstroke to the time when the potential returned to 30, 50, and 90% of the AP amplitude.

To compare goodness of fit between APs, we calculated error terms as the sum of squared differences (SSD):

(1) $$\text{SSD = }\overset{t_{\max}}{\underset{t=t_{0}}{\sum }}{\left[V_{1}\left(t\right)-V_{2}\left(t\right)\right]}^{2}$$

where *V*_1_ and *V*_2_ represent the transmembrane potential from the two trials to be compared (model vs. experimental APs, or APs with CTC on vs. off). In the case of experimental data, average waveforms were used. The start time (*t*_0_) was defined as the time of crossing of −40 mV on the AP upstroke to avoid artifactual errors associated with the stimulus foot potential. The duration of the voltage segment for error calculation (*t*_max_) was set to 300 ms, as previous tests using an entire AP cycle (1000 ms) did not provide better fits.

For statistical analysis, we used the Student’s *t*-test for unpaired data with equal variance; by conventional criteria, *p* < 0.05 is considered statistically significant.

### CTC circuit

The CTC circuit was described in detail in a previous publication (Ahrens-Nicklas and Christini, 2009). Briefly, a real cell (target myocyte) is simultaneously coupled to a target-canceling computational model (a mathematical model of the target cell) and a recipient computational model (a mathematical model of the desired cell-type, i.e., human or guinea pig), in a closed-loop circuit (Figure 1). Specifically, in step 1, the target cell voltage is measured and simultaneously input to the recipient and target-canceling models (step 2). The total transmembrane current calculated for each model (*I*_cancel_ and *I*_recipient_) is scaled by the ratio of the target cell capacitance to the model cell capacitance (*K*_*c*_ and *K*_*r*_; step 3). Their difference current, *I*_diff_, is calculated by subtracting the scaled target-canceling model current from the scaled recipient model current (step 4). Stimulus current and the patch-clamp seal leak current are added to the difference current to produce the injected current (step 5). Lastly, this current is injected into the target cell (step 6). Thus, for each cycle of the circuit, a time-dependent current is inserted into the mouse myocyte to compensate for the difference between mouse myocyte and recipient model cell currents.

### Computational models

To model the target cell AP, we used the Pandit et al. model modified for a ventricular neonatal mouse cardiomyocyte (CellML, 2001; Pandit et al., 2001; Henriquez et al., 2004; Tranquillo et al., 2005). We used the same modifications as in Ahrens-Nicklas and Christini (2009), except that the sodium conductance (*g*_Na_) was decreased to 0.8 μS (as in Pandit et al., 2001; Henriquez et al., 2004; Tranquillo et al., 2005) to match our recorded AP overshoot. Ionic concentrations were kept at their default values ([K^+^]_o_ = 5.4 mM, [Na^+^]_o_ = 140 mM, [Ca^2+^]_o_ = 1.2 mM; [Na^+^]_i_ = 8.6 mM, [K^+^]_i_ = 142 mM, [Ca^2+^]_i_ = 0.079 μM), which are close to the experimental conditions.

For the CTC recipient model cell, we used the Faber–Rudy guinea pig ventricular myocyte model (Faber and Rudy, 2000) or the reduced ten Tusscher–Panfilov human ventricular myocyte model (ten Tusscher and Panfilov, 2006).

Numerical integration of the recipient models (Faber–Rudy or ten Tusscher–Panfilov) was done using a forward Euler scheme with a time step of 0.01 ms, while for the neonatal mouse model (modified Pandit) the time step was 0.1 ms. To speed up integration we used a Real-Time Math library that permits fast approximations to compute exponentials and power exponents [i.e., the *exp()* and *pow()* functions; Schraudolph, 2007; Vinyals et al., 2007].

### Template-based model selection

As a first approach to tune the modified Pandit et al. (2001) model to our recorded neonatal mouse APs, we developed a suite of nine models yielding APD_80_ values ranging from 40 to 120 ms in 10 ms increments, by varying the steady-state potassium conductance (*g*_ss_) and the slowly inactivating potassium conductance (*g*_Kslow_; CellML, 2001; Ahrens-Nicklas and Christini, 2009; see Figure S1; Table S1 in Supplementary Material). With this approach, the model with the APD_80_ value closest to that of the real myocyte is selected and used to simulate the target-canceling currents when applying CTC to that myocyte (as in Ahrens-Nicklas and Christini, 2009).

### Genetic algorithm model tuning

As an alternative to the template-based model selection to obtain a target-canceling model that fits a particular target myocyte, we investigated a GA to modify dominant conductances of the neonatal mouse model. The GA was previously implemented in our laboratory (Kherlopian et al., 2011), based on Sastry, 2007. Here, it was modified to run sufficiently fast to fit a model to a living cell during an experiment.

**Figure 2:**
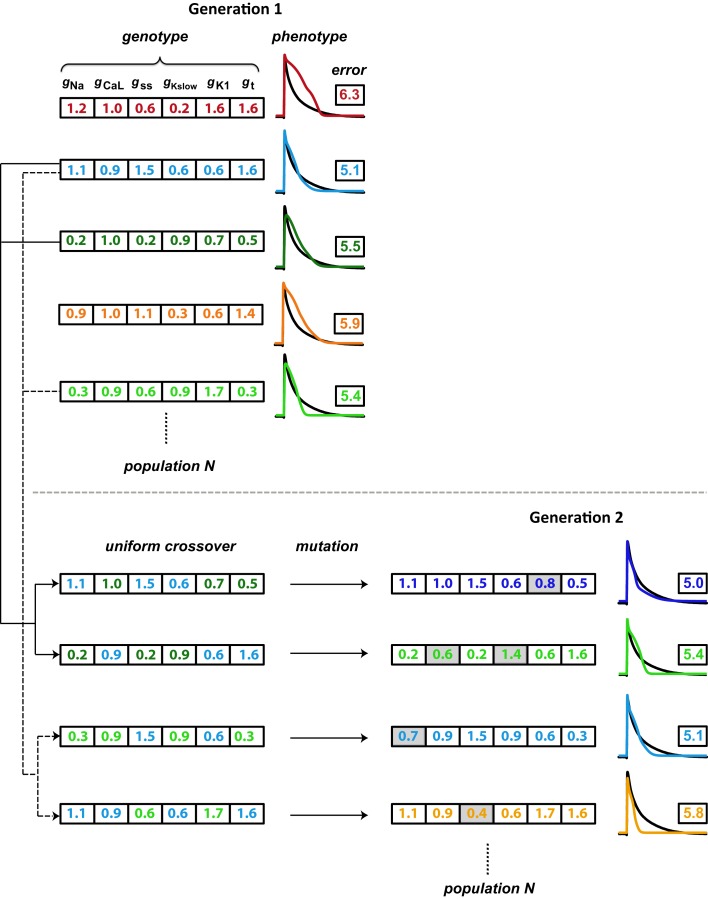
**Schematic illustration of the genetic algorithm processes**. Each individual is represented by a color and consists of a genotype (here, scaling factors for six conductance parameters), which results in a phenotype (simulated AP). The phenotype is compared to the objective AP (black trace) through an error value (the logarithm of the sum of squared differences). Individuals with lower errors are most likely to be chosen as parents for the subsequent generation. As the first step in generating offspring, two parents each contribute different parts of their genotype in a process known as crossover, to generate two children whose genotypes consist of values inherited from their parents. A second step introduces variation (mutations; gray shading) in these gene values. Finally, the offspring phenotype and associated error value are evaluated. These processes of parent selection, crossover, mutation, and offspring generation and evaluation are repeated to obtain the desired number of individuals in the subsequent generation. In our optimizations, the GA cycles through 15 generations.

The GA technique (Figure 2) is based on principles from evolutionary biology, such as selection (higher probability for parameters from model instantiations with good fits to persist in optimization), crossover (combining parameters from model instantiations with good fits), and mutation (perturbing individual parameters within a model instantiation to maintain diversity in the population of candidate solutions). The GA progresses in generations by changing selected parameters, a set of which define the genotype of an individual. An individual represents a model instantiation, and the simulated membrane potential constitutes the phenotype. Within each generation, we compare the phenotype of each individual to the optimization objective (the average experimental neonatal mouse AP) through an error defined as the SSD (Equation 1). A flow chart of the GA algorithm is shown in Figure S3 in Supplementary Material.

We used the following settings of configurations and parameters in the GA toolbox of Sastry (2007). For the selection of parents that form the basis of individuals in the subsequent generation, we used tournament selection without replacement. In this method, two individuals are selected at random from the population and the one with a higher fitness (lower error) goes into the mating pool and becomes a parent. Looping through all individuals once thus creates a mating pool half the size of the generation size. The selection process is then cycled through a second time such that the number of parents in the mating pool equals the number of individuals in each generation (Figure S3). Parents in the mating pool are then paired sequentially from the random order in which they won tournaments. Each parent pair produces two children. Most parent pairs undergo crossover (the crossover probability was set to 0.9), in which their parameter values are swapped in the progeny (Figure 2). We used the simulated binary crossover (SBX) technique with polynomial order 10 and single parameter genewise swap probability of 0.5. After crossover, parameter values may undergo mutation (Figure 2). In addition, parents that do not undergo crossover instead become their own children and may be mutated. To induce mutations, we used a polynomial mutation operator with order 20, which was centered on individuals’ current parameter values. The mutation probability was set to 0.1 per gene. Finally, we applied the elitism strategy in which the most fit individual within each generation is directly copied into the subsequent generation, replacing the least fit one.

GAs are typically computationally expensive, given that an entire population of candidate solutions must be evaluated over many generations. To enable rapid fitting of a living cell, we focused on reducing the runtime of evaluating individual candidate solutions as well as reducing the total number of candidate solution evaluations required to reach strong fits. To shorten the numerical integration time of candidate solutions we adhered to an efficient model of relatively low complexity (CellML, 2001; Pandit et al., 2001; Henriquez et al., 2004). We found that restricting the parameter space as described below, limiting the generation size to 40 individuals, and restricting the evolution to 15 generations decreased the number of candidate solutions sufficiently for the GA to reach a low-error solution within a short amount of time (13–15 s).

To restrict the size of the parameter space, the optimization was limited to scaling six main conductance parameters, each of which was constrained within a search range. The six conductances were those corresponding to the six largest currents in the unperturbed model: *g*_Na_, *g*_ss_, *g*_Kslow_, L-type calcium channel conductance (*g*_CaL_), inwardly rectifying potassium conductance (*g*_K1_), and the Ca^2+^-independent transient outward K^+^ conductance (*g*_t_). As a starting point for the GA optimization, we selected the neonatal mouse model with an APD_80_ of 60 ms (Table S1) and set the conductance search ranges as: *g*_Na_, *g*_K1_, *g*_t_: ±90%; *g*_CaL_: ±10%, and *g*_ss_, *g*_Kslow_:−90 to +200% around their values in that model. This combination of conductances and search ranges allows the GA to converge to neonatal mouse models that explore various AP shapes and durations, with APD values from 40 to 160 ms.

## Results

**Figure 3:**
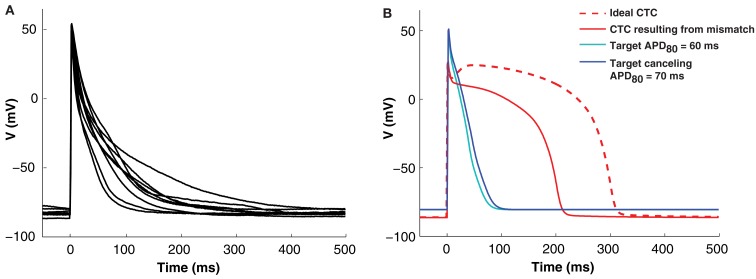
**Cell-to-cell variability and target to target-canceling model mismatch**. **(A)** Average APs (10 successive beats) from nine neonatal mouse myocytes demonstrating morphological cell-to-cell AP variation. **(B)** *In silico* APs obtained using the same neonatal mouse model (the neonatal mouse model with an APD_80_ of 60 ms; cyan) in both the target and target-canceling positions produce an ideal conversion to the human recipient model (red, dashed line). However, when a small discrepancy is simulated by perturbing the target-canceling model (to neonatal mouse model with an APD_80_ of 70 ms; blue) there is imperfect anthropomorphization with insufficient AP prolongation (red, solid line).

Isolated neonatal mouse ventricular myocytes exhibit significant variability in AP morphology and duration (Figure 3A). Such cell-to-cell variability makes matching between a target cell and the CTC target-canceling model difficult. However, without a good fit, CTC performance is impaired. This is illustrated in Figure 3B, where we show a simulation of a CTC run with a small discrepancy (10 ms APD difference) between the target and the target-canceling model. In this case, the difference leads to an inability of CTC to appropriately prolong the target AP, shown in a simulated trial by using the same model for the target and its canceling model.

### Genetic algorithm progression and model optimization

**Figure 4:**
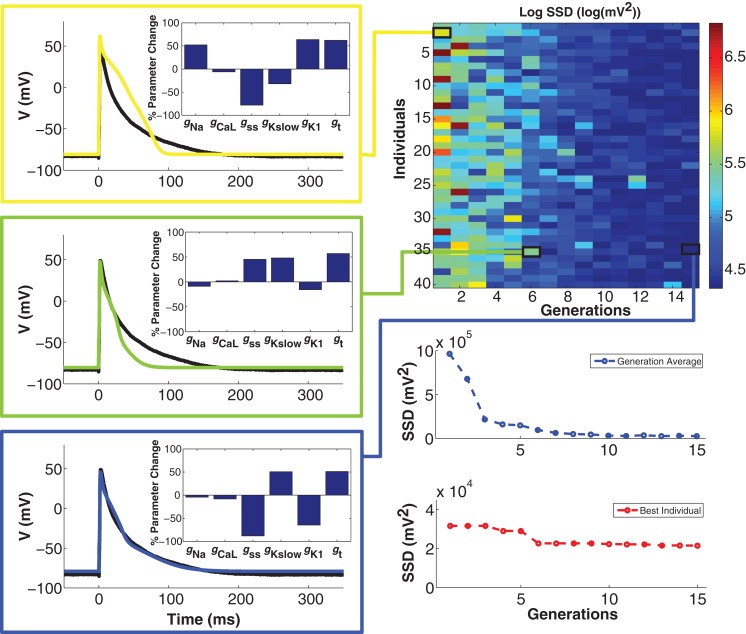
**Genetic algorithm model optimization**. Top right panel: population of 40 individuals evolving over 15 generations; color bar denotes value of the error (sum of squared differences; SSD) between model and experiment AP. With GA progression, high-error individuals become less frequent and low-error individuals start to dominate. Left panels: examples of genotypes (scaling factors for conductance parameters as percent change from unperturbed model parameters; insets) and corresponding phenotypes (color-coded as per error heat map) with GA progression. In the first generation (yellow), there is a substantial difference between the phenotype and the optimization objective (black trace). In the sixth generation (green), a very different genotype gives a better fit. In the 15th and final generation (blue), another genotype gives a very strong fit. Bottom right panels: convergence of the average error for the population ensemble (blue) as well as the error of the best individual (red) occurs within a few generations.

To minimize cell model mismatch, we investigated a GA optimization to fit a model to each target cell. An example of a GA-fit to a real cell AP is shown in Figure 4. The first generation of individuals in the GA was set by random sampling from a uniform distribution for the six model conductances. This results in a relatively large error (Figure 4, right), as the random parameter selections produced APs with morphologies different from the objective (Figure 4, top left). With GA progression, error values decrease as fits become better. An individual from the sixth generation (Figure 4, middle left) produces a phenotype that is closer to the objective, while an individual in the 15th generation produces a strong fit (Figure 4, bottom left). Note that the parameter combinations giving rise to these different model instantiations vary substantially (insets in Figure 4, left). The GA converges in relatively few generations, as seen in the error for both the generation average and for the best individual solution in each generation (Figure 4, bottom right), as well as in the conductance parameter values (Figure S2 in Supplementary Material). When using GA in conjunction with CTC, we used the lowest-error individual from the 15th generation as the optimal cell-specific model fit.

### CTC experiments with guinea pig recipient model

To test the performance of the CTC with a GA-optimized target-canceling model, we investigated the ability of CTC to transform *in vitro* neonatal mouse ventricular myocyte APs to mimic characteristic guinea pig ventricular APs. For comparison, we applied the CTC without GA optimization, using instead a nominal target-canceling model selected based on APD from a range of candidate models as described in Methods (see Template-Based Model Selection).

**Figure 5:**
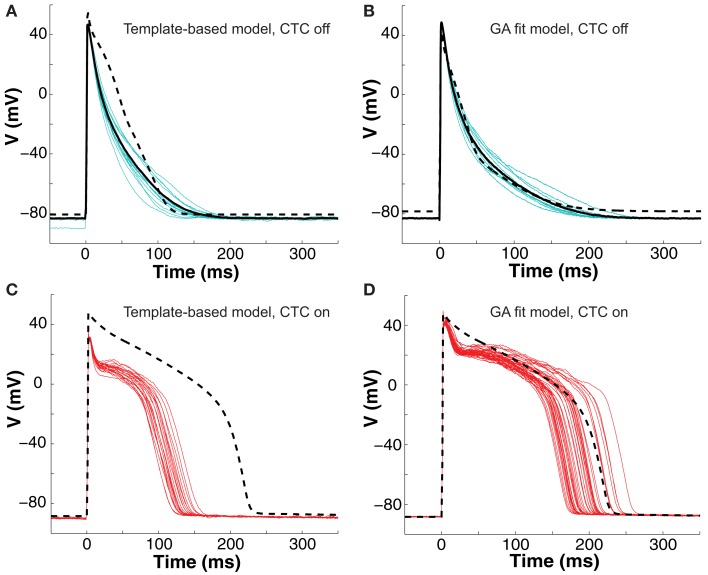
**Neonatal mouse APs converted to guinea pig AP**. **(A)** Recordings of ten consecutive APs from an isolated neonatal mouse myocyte (cyan traces) are averaged (solid black line). The nominal model (dashed line) is selected from a suite of nine candidate models based solely on the closest match to its APD_80_ value, but fit the early part of the AP poorly. **(B)** We then recorded ten successive APs from the same cell (cyan traces) and use their average (solid black line) as the optimization objective. The GA returns a close fit (dashed line). **(C)** With CTC on, template-based target-canceling model selection resulted in APs (red traces; 20 subsequent APs) morphologically similar to the recipient guinea pig model AP (dashed line), but of much shorter duration. **(D)** Applying CTC using the GA-optimized model gave guinea pig-like action potentials (red traces; 40 subsequent APs) that mimic the recipient cell model prediction (dashed line).

A representative example of a cell undergoing CTC with and without GA optimization is shown in Figure 5. Because of considerable beat-to-beat variability in the experiment recordings (cyan traces in Figures 5A,B), we use an average of ten consecutive APs (black solid lines) as the basis for either selecting or optimizing the target-canceling model. Although the selected nominal model (dashed trace in Figure 5A) has an APD_80_ close to the average experiment value, the AP morphology is very different, causing significant mismatch between the nominal model and the recorded data. In comparison, the GA-optimized model (dashed trace in Figure 5B) fits the waveform closely.

With CTC on, APs from the isolated neonatal mouse myocyte were transformed to become more like those of the guinea pig model (Figures 5C,D). The CTC both increased APD and induced the expected plateau, intrinsically absent in the murine AP. However, the template-based model selection CTC produced guinea pig-like APs that were far from the model-predicted shape (Figure 5C). In contrast, recordings from the same cell using the GA-optimized model produced transformed APs that accurately mimicked those of the recipient model (Figure 5D).

**Figure 6:**
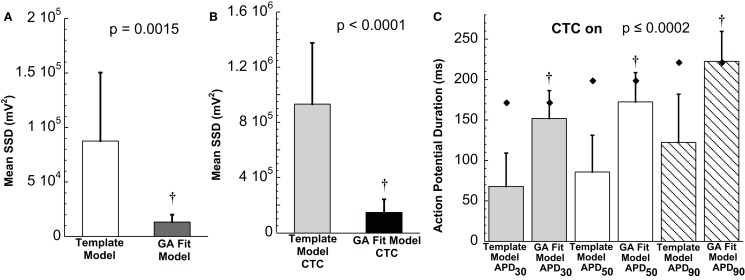
**Statistical analysis of target cell matching and CTC performance with guinea pig recipient model**. **(A)** Average error (SSD) between unperturbed *in vitro* neonatal mouse myocyte APs and either selected template-based model or GA-fit model showing better fits with GA optimization. **(B)** Average error for CTC-on APs using the guinea pig recipient model demonstrating higher CTC accuracy with GA-optimized models over template-based model usage. **(C)** AP duration at different repolarization levels for template-based model CTC vs. GA-fitting CTC. For all three repolarization markers, the GA-fit model CTC reproduces recipient model APD values (diamonds), while template-based model CTC produces waveforms of insufficient duration. Error bars in all panels give standard deviation, *n* = 10 cells.

The ability of the GA to provide a much better fit to a particular neonatal mouse myocyte AP than the template-based model selection was seen consistently in all recordings (*n* = 10). The average error between model and experimental waveform was significantly smaller for the GA-fit model than for the nominal models (Figure 6A). Likewise, the increased CTC accuracy with GA-optimized models compared to template-based model selection was consistent across the myocyte population, with significantly lower errors between recipient and target APs for GA-fitting CTC (Figure 6B). With template-based model selection prior to CTC, the discrepancy between target myocyte and recipient waveforms was due mainly to APs being too short in the target myocytes (Figure 6C). In contrast, GA optimization resulted in converted APs with durations at different repolarization levels matching those of the desired recipient model (Figure 6C).

### CTC experiments with human recipient model

**Figure 7:**
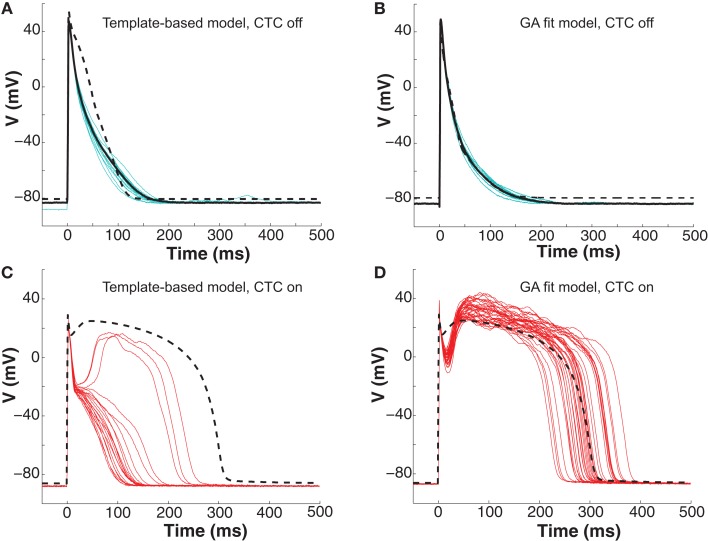
**Neonatal mouse APs anthropomorphized to human AP**. **(A)** Ten consecutive neonatal mouse myocyte APs (cyan traces) recorded *in vitro*, their average (solid black line), and the APD_80_-based nominal model (dashed line). **(B)** Ten APs from the same murine myocyte (cyan traces), their average (solid black line), and the GA-optimized model (dashed line). **(C)** With template-based model CTC (red traces), anthropomorphization frequently failed in forming a dome and prolonging the AP to the extent of the recipient model (dashed line). **(D)** With GA optimization, CTC successfully prolonged the mouse AP, induced a plateau phase, but exaggerated the notch.

**Figure 8:**
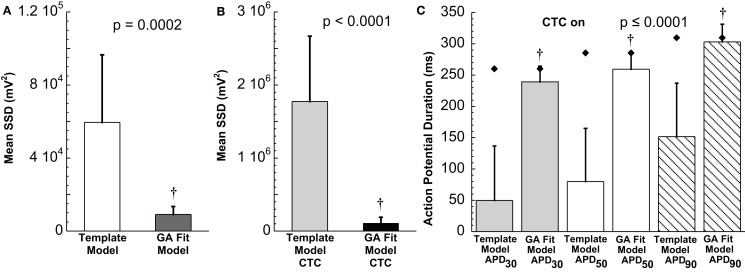
**Statistical analysis of target cell matching and CTC performance with human recipient model**. **(A)** Decrease in average error between neonatal mouse myocyte APs and GA-fit models compared to nominal models demonstrating better matching with GA optimization. **(B)** Average error for CTC-on APs using the human recipient model showing better CTC performance with GA-optimized models over template-based model selection. **(C)** AP duration at different repolarization levels for template-based model CTC vs. GA-fitting CTC. For all three repolarization markers, GA-fit model CTC reproduces recipient model APD values (diamonds), while template-based model CTC resulted in too short waveforms. Error bars in all panels give standard deviation, *n* = 11 cells.

To further investigate CTC performance, we subjected neonatal mouse cells to another cross-species transformation by using a human ventricular myocyte model as the recipient. In this myocyte population, GA optimization again provided a much better fit of the target-canceling model to the recorded data than did template-based model selection (Figures 7A,B and 8A).

Early repolarization is much faster in the human model AP compared to the guinea pig model, with the human AP having a characteristic notch-and-dome morphology. When using the human recipient model in CTC in conjunction with GA optimization, neonatal mouse APs were again converted into prolonged APs with a sustained plateau, but had an exaggerated notch-and-dome morphology (Figure 7D). Despite this difference between the transformed APs and the recipient model in the early phases of the AP, the duration of the anthropomorphized APs matched those of the recipient model (Figures 7D and 8C).

In contrast, using template-based model selection, CTC was less successful. In many trials, the converted AP failed to develop the desired dome (Figure 7C). This resulted in large discrepancies between CTC-on APs and the recipient model AP (Figure 8B), seen also as a severe shortening in APD, especially in early repolarization (APD_30_ and APD_50_; Figure 8C). We studied a total of 11 cells using the human recipient model. Of those, template-based model CTC failed to develop AP domes in some cycles in six cells, while GA optimization CTC failed in only one myocyte. These dome vs. no-dome dynamics in template-based model CTC resulted in high APD variability (error bars in Figure 8C).

### Target and target-canceling model mismatch decreases CTC accuracy

It is clear from the experiments described above that the ability of the CTC to match the recipient model AP requires a close fit between the target myocyte and the target-canceling model. We now turn to an analysis of the CTC circuit currents to provide mechanistic insights as to why CTC fails to prolong the target myocyte AP sufficiently when using template-based model selection.

**Figure 9:**
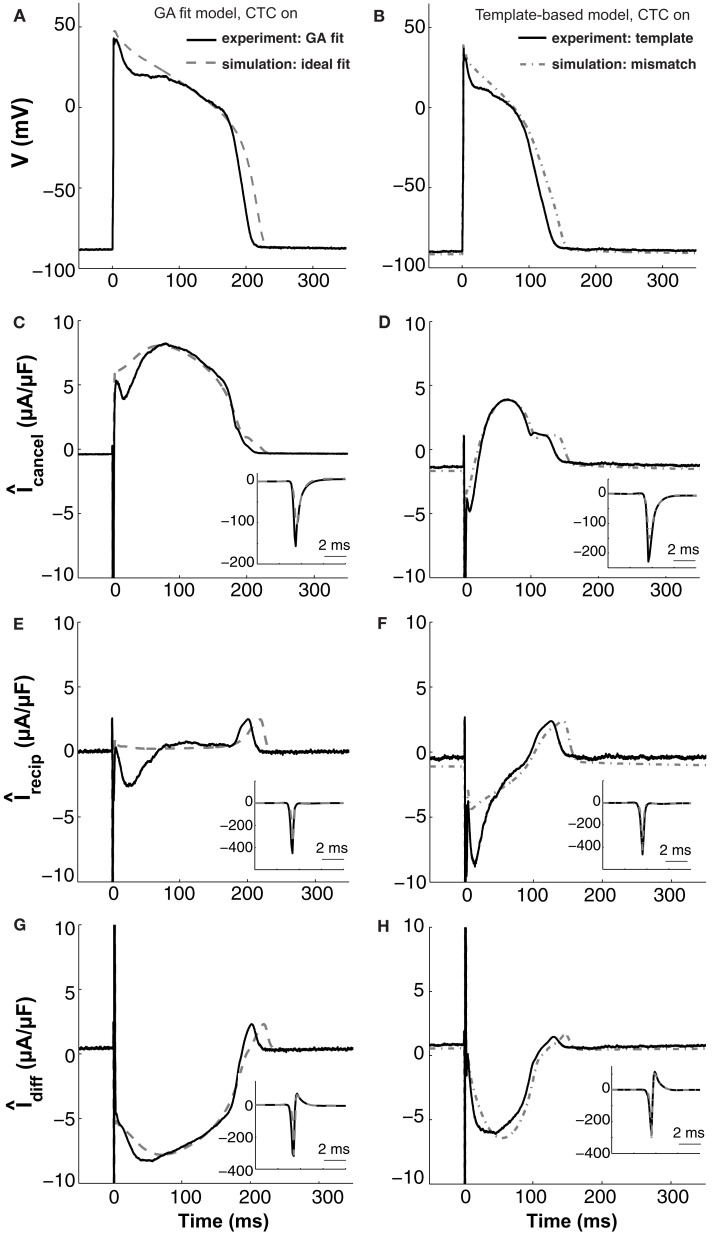
***In silico* and *in vitro* CTC circuit currents**. Left panels show APs and related currents in CTC for an experiment using a GA-optimized target-canceling model (solid traces) as well as the corresponding ideal case of simulating that optimized model as both the myocyte and its canceling model (dashed traces). Right panels compare CTC-on APs and currents for an experiment using template-based model selection for the target-canceling model (solid traces) to an *in silico* case of mismatch between that same template-based target-canceling model and a GA-optimized model to simulate the target myocyte (dashed-dot traces). **(A)** and **(B)** target myocyte/model AP; **(C)** and **(D)** total current in the target-canceling myocyte/model; **(E)** and **(F)** total current in the recipient guinea pig model; **(G)** and **(H)** their difference current. With the GA optimization, the currents are close to their ideal behavior and AP transformation is accurate. When template-based selection is used, a lack of repolarizing current in the target-canceling model to counter that in the real myocyte leads to improper repolarization of the target myocyte. Insets show initial peak-current profiles. Same cell as used in Figure 5.

To assist the analysis, we simulate an ideal situation where the target-canceling model captures the target cell AP exactly, by having the exact same membrane currents. We do so by utilizing a GA-optimized model as both the target and the target-canceling cell (dashed gray traces in Figure 9, left). The simulated CTC-transformed AP (to a guinea pig recipient model) for this ideal situation is shown in Figure 9A. During this AP, the simulated current in the recipient cell model undergoes its characteristic changes: large inward spike during the upstroke, tiny net outward current during slow repolarization in phase two, and broad outward current peak during the rapid repolarization of phase three (Figure 9E). During phase two, a large outward current is induced in the target-canceling model (Figure 9C), which is expected for a murine model with rapid intrinsic repolarization. Because *Î*_diff_=*Î*_recipient_–*Î*_cancel_, where *Î* indicates capacitance-normalized current, a large outward target-canceling current and a small recipient model current result in a large inward difference current being injected into the target cell model (Figure 9G), sustaining phase two of the CTC-transformed AP.

These *in silico* CTC currents and AP are quite similar to those obtained experimentally during CTC (solid traces in Figure 9, left) when using that same GA-optimized target-canceling model, which was obtained as a fit to the AP recorded from this particular cell (same cell as in Figure 5). However, relative to the *in silico* case, in the experiment there is less outward current in the target-canceling model immediately after the AP upstroke (Figure 9C). This small lack of outward current is consistent with some early repolarization in the target myocyte (Figure 9A) and hence in the recipient model. This repolarization, in turn, triggers an inward current in the recipient model (Figure 9E), which helps create a difference current close to the ideal (Figure 9G).

This comparison between *in silico* and experimental CTC currents suggests that GA optimization provides a well-fit target-canceling model. We now use a similar comparison to analyze CTC currents during template-based model selection (Figure 9, right).

With template-based model selection (solid traces in Figure 9, right), there is a large mismatch between the ideal (Figure 9C) and the recorded target-canceling current (Figure 9D). Instead, the CTC currents and the anthropomorphized AP are well-captured by a simulation that mimics the experimental situation closely: a deliberate mismatch between the target and its canceling model was induced by using template-based model selection for the target-canceling model and the GA-optimized model for the myocyte (dashed-dot traces in Figure 9, right). In both this simulation and the experiment, the target-canceling current is much smaller than in the ideal case, and is even inward instead of outward early on during the AP (Figure 9D). This results in an inadequate difference current (Figure 9H) despite an increased inward recipient model current (Figure 9F), and in an inability for CTC to sustain a sufficiently depolarized plateau (Figure 9B). Consequently, if the target-canceling model does not provide a large enough outward current early on during the AP, uncompensated outward current in the myocyte will repolarize it and result in deficient conversion.

Thus, this comparison between template-based model selection vs. GA optimization for CTC experiments corroborates the hypothesis that mismatch between a target cell and the target-canceling model causes imperfect CTC. In addition, the comparison illustrates in particular how a lack of outward current in the target-canceling model leads to insufficient AP prolongation in CTC.

## Discussion

The mouse serves as a ubiquitous mammalian animal model in biological research, in part due to our ability to manipulate its genome. Indeed, genomic resources for the mouse (Genome Reference Consortium, 2012) are rapidly increasing, and CTC can be added as a new tool to screen for phenotypes in excitable cell dynamics, e.g., arising from mutations or polymorphisms in genes encoding for ion channels or for ion channel regulatory proteins. In cardiac arrhythmia research, AP transformations across species can help overcome the inherent difficulties in translating murine electrophysiological and pathophysiological traits into relevant human counter properties.

In this study, we demonstrated that the AP waveform of a ventricular neonatal mouse myocyte can be converted into that of a ventricular guinea pig or human myocyte AP, in real-time, through the CTC technique. We presented a method to efficiently provide a model of a living cell, based on a GA optimization. We further demonstrated that creating such a cell-specific model improves the ability of CTC to anthropomorphize the murine myocyte AP.

### Use of template-based model selection in CTC

For CTC to work, the experimental current recorded in the target myocyte must be of the same amplitude and exhibit the same time dependence as its theoretical model correspondent. Selecting a CTC target-canceling model out of a suite of mouse models based on APD_80_, as in our previous study (Ahrens-Nicklas and Christini, 2009), offers a limited AP matching to a given recorded neonatal mouse AP. Moreover, two neonatal mouse cells can have the same APD_80_ values but still differ in AP morphology. Hence template-based model selection could almost certainly be improved by considering the entire AP waveform. However, to change the AP shape requires varying more conductance parameters than the two originally chosen for this procedure. In turn, adding more parameters to be changed systematically would dramatically increase the number of template models in the suite, which would increase the search time for the best template.

Neonatal mouse cells undergo rapid developmental changes (Grandy et al., 2007). In our current recordings, using mouse myocytes at a different developmental age compared to our previous study, our recordings consistently had faster initial repolarization than the nominal models. Our simulations of intended mismatches between target myocyte and target-canceling model suggest that in CTC, a lack of repolarization current in the target-canceling model results in uncompensated outward current in the target myocyte, which repolarizes it too quickly (Figure 9). Inversely, excess repolarization current in the target-canceling model cause APs to be longer than those of the recipient model when simulating CTC (not shown).

When using the human ventricular myocyte recipient model and template-based model selection, target myocytes were frequently rapidly repolarized to a voltage range from which they would either depolarize and form a characteristic dome or fully repolarize (Figure 7). Such dome vs. loss-of-dome dynamics can depend very sensitively on the magnitude of the total current available after the initial repolarization (Maoz et al., 2009). Hence, although the current injected into the target cell with CTC may not vary much on a beat-to-beat basis, it can result in very different AP morphologies and APD values.

### GA optimization and limitations

While different types of optimization techniques (such as GAs, simulated annealing, and gradient descent methods), have been tested and compared for neuronal models (Vanier and Bower, 1999), few studies have investigated optimization of cardiac electrophysiology models. One such study applied a GA to fit a cardiac cell model (Syed et al., 2005), while others used simulated annealing and/or simplex algorithms to fit ionic current data (Iyer et al., 2004; Moreno et al., 2011).

The error landscape that an optimization method must traverse is dependent on the objective function as well as on the dynamics and parameter dependence of the model. Due to the complexity of model behaviors in parameter space (Achard and De Schutter, 2006; Taylor et al., 2006), an error landscape can contain multiple local minima. The main advantage of the GA over traditional optimization methods such as gradient search is that lower fitness solutions can be selected as the GA progresses. Examples of this can be seen in Figure 4, where high-error phenotypes emerge, e.g., from individual number 24 in generation 12 and individual number 40 in generation 14. Such reversion can allow the GA to escape local minima in the error landscape. This ability comes at the cost of increased computation times as larger regions of parameter space are explored.

Due to the severe experiment time constraints when fitting a living cell, we restricted the generation size to 40 individuals and the number of generations to 15. Using fewer than 40 individuals lead to loss of diversity, which in turn can cause the GA to get stuck in a local minimum unless the mutation rate is high. Conversely, increases beyond 40 individuals lead to prolonged simulation time with no gain in error performance in the final generation. Testing up to 60 generations, we also found that extension beyond 15 generations led to very minor error improvement in the best individual. We limited the parameters to be varied within the GA to six key conductance parameters and thus did not allow for variation in ion channel kinetics. Finally, we limited the objective to a single AP, which was sufficient to generate a close AP match at the given pacing rate. To create a computational model capable of fitting broader dynamics such as APD rate dependence would likely necessitate using a longer objective including APs at different pacing rates.

Despite these constraints in the GA, for all target myocytes tested, we were able to obtain fits that fell within the naturally occurring beat-to-beat AP variability, except that the resting membrane potential was underestimated by a few millivolts in most cases. However, CTC correctly compensated for this difference such that anthropomorphized murine myocytes had similar resting membrane potentials as the recipient model cells (Figures 5 and 7).

The GA consistently converged to solution models that had increased values of *g*_t_, the conductance of the transient outward current. An increase in this current causes more rapid early repolarization and is consistent with the experimentally recorded APs undergoing faster initial repolarization than the nominal model. Thus, the GA provides a better target-canceling model and ensures more accurate CTC performance.

### CTC limitations

Because of the intrinsic beat-to-beat variability in the target cell AP and its underlying currents, there is an unavoidable mismatch between the target cell currents and the target-canceling model currents on a beat-to-beat level. This introduces an error when using CTC, with individual transformed APs not necessarily matching that of the recipient model. We found however that when applying CTC to tens of APs, the average duration of transformed APs fit the desired APD, when the average mismatch is first reduced by GA optimization.

The amplitude of the current injected into a myocyte in CTC is scaled to its measured capacitance. Hence, if the capacitance is not determined accurately, the injected current will not reflect the intrinsic current correctly and anthropomorphization errors similar to those stemming from using an ill-fitting target-canceling model arise.

Our experiments were performed at room temperature, while computational models are developed toward physiological temperature. Despite the differences in kinetics associated with this temperature discrepancy, the GA was able to match the experimentally recorded waveforms well.

Although CTC is capable of inducing a sustained plateau and prolonging the APD, CTC does not recapitulate phase one of the recipient model AP well. This is particularly clear when using the human recipient model, in which case the notch-and-dome morphology is magnified. Hence, CTC is more useful for studying later parts of the AP, which are also more typically involved in arrhythmogenesis.

In parallel with the distinctive APs, the intracellular calcium cycling dynamics differ between murine and human ventricular myocytes (Gao et al., 1998; Bers, 2008). Because the CTC does not compensate for differences in calcium transients, it is likely that a CTC controlled murine cardiac myocyte undergoes calcium dynamics different from that in human myocytes. Calcium imaging during CTC experiments could help shed light on this.

### Perspectives and other applications of GA and CTC

The during-experiment GA-fit may be applied to other excitable cells. Neuronal dynamics have been investigated previously using evolutionary strategies, optimizing models of pre-recorded cell or channel activity (Achard and De Schutter, 2006; Druckmann et al., 2007; Hendrickson et al., 2011). However, spike-time variability, spatial non-uniformity, and non-uniqueness of model solutions all present obstacles to living-neuron GA-fitting.

While we have chosen here to transform AP waveforms from one species to another, CTC would also be useful for transforming APs between other variant cell-types, in an effort to quantify underlying current differences. Such cell transformations could include cells from different regions of the heart or cells at different developmental stages. As done in our previous study (Ahrens-Nicklas and Christini, 2009), cell transformations may be carried out in combination with ion channel block to investigate cell-type variations in ionic currents.

In summary, the CTC allows the murine myocyte to undergo human-like membrane potential dynamics in current-clamp mode. It is suitable for multiple electrophysiological applications, including studying effects of genetic variations and screening drug compounds.

## Conflict of Interest Statement

The authors declare that the research was conducted in the absence of any commercial or financial relationships that could be construed as a potential conflict of interest.

## Supplementary Material

The Supplementary Material for this article can be found online at http://www.frontiersin.org/Computational_Physiology_and_Medicine/10.3389/fphys.2012.00421/abstract
